# Cognitive bias in rats is not influenced by oxytocin

**DOI:** 10.3389/fpsyg.2015.01306

**Published:** 2015-09-02

**Authors:** Molly C. McGuire, Keith L. Williams, Lisa L. M. Welling, Jennifer Vonk

**Affiliations:** Department of Psychology, Oakland University, Rochester, MI, USA

**Keywords:** cognitive bias, rats, oxytocin, ambiguous cue, place preference, approach latency

## Abstract

The effect of oxytocin on cognitive bias was investigated in rats in a modified conditioned place preference paradigm. Fifteen male rats were trained to discriminate between two different cue combinations, one paired with palatable foods (reward training), and the other paired with unpalatable food (aversive training). Next, their reactions to two ambiguous cue combinations were evaluated and their latency to contact the goal pot recorded. Rats were injected with either oxytocin (OT) or saline with the prediction that rats administered OT would display a shorter average latency to approach on ambiguous trials. There was no significant difference between latencies to approach on ambiguous trials compared to reward trials, but the rats were significantly slower on the aversive compared to the ambiguous conditions. Oxytocin did not affect approach time; however, it was unclear, after follow-up testing, whether the OT doses tested were sufficient to produce the desired effects on cognitive bias. Future research should consider this possibility.

## Introduction

In humans, cognitive bias describes the influence of emotional states on biases in information-processing (Bethell et al., 2012). Specifically, a negative cognitive bias describes the tendency to process information that should have a neutral valence as negative (i.e., to be pessimistic). Many non-human species have also provided evidence of existing cognitive biases. For example, there is increasing evidence that the internal affective state of an animal can influence their reactions to ambiguous stimuli (Burman et al., 2009). A stimulus (e.g., visual or auditory cue) is considered ambiguous if it is novel and distinct from discrete trained cues, or is a novel combination of elements of the trained conditions. Reactions to an ambiguous and novel stimulus should differ depending on the animal’s existing bias or their present internal state as a function of the context when encountering the stimulus. For example, an animal in a negative internal state (e.g., fear, anxiety, or stress) would be more likely to interpret an ambiguous stimulus as negative or threatening and would respond accordingly. Alternatively, an animal in a positive internal state (e.g., relaxation, reward, or playfulness) would be more likely to respond to the same ambiguous stimulus in a positive manner. By assessing the reaction to such novel, ambiguous stimuli researchers hope to gain insight into the affective states and mental welfare of animals (Bethell et al., 2012). The most commonly used technique when investigating cognitive bias in non-humans is a go/no-go task in which the animals are trained to discriminate between two distinct cues, one that they are required to give a specific response to (for example, in order to gain a reward), and the other that they are required to withhold a response to avoid an unpleasant stimulus. Researchers then present the animal with ambiguous cues, which are typically intermediate between the trained cues, or completely novel and distinct (Bethell et al., 2012; Briefer and McElligott, 2013).

These ambiguous cues are often presented immediately following environmental manipulations aimed to invoke negative or positive affective states (e.g., stress-provoking versus enriching events). An animal can be described as behaving in an optimistic fashion (i.e., demonstrating a positive cognitive bias) if it responds to an ambiguous item in the same manner as to those items previously associated with reward. On the other hand, an animal can be described as behaving pessimistically (i.e., demonstrating a negative cognitive bias) if it responds in the same manner as it did to an item associated with something aversive (e.g., something punitive or simply the lack of reward).

Another common measure of cognitive bias in animals is the latency to approach an ambiguous stimulus after exposure to negative or positive treatment conditions (Boissy et al., 2007; Burman et al., 2011; Destrez et al., 2012; Briefer and McElligott, 2013). After experiencing presumably negative experiences, rats and pigs approached an ambiguous stimulus significantly more slowly than they did after being exposed to a putatively positive experience (Burman et al., 2009; Douglas et al., 2012). In this case, a negative cognitive bias is defined as a slower latency to approach a novel stimulus, whereby it is assumed that the animal is demonstrating an expectation of negative outcome or anxiety. When rats were transitioned from preferred lighting conditions (low light) to aversive lighting conditions (high intensity lighting) they traveled more slowly to ambiguous sites compared to rats that had transitioned from aversive lighting to preferred lighting or even rats that remained in aversive lighting throughout testing (Burman et al., 2009). Because there was no significant difference in how quickly any of the rats approached known reward locations, the researchers concluded that the lighting conditions were influencing the cognitive biases of the rats rather than speed of movement in general. Cognitive biases have also been examined in various other animal species, including pigs (Douglas et al., 2012), dogs (Burman et al., 2011), grizzly bears (Keen et al., 2014), and goats (Briefer and McElligott, 2013).

Much of the existing research on cognitive bias has focused on external influences such as long-term environmental manipulations, such as changing enrichment levels (Bateson and Matheson, 2007; Douglas et al., 2012). More recently, researchers have begun to investigate pharmacological manipulations, attempting to influence cognitive biases by directly manipulating mechanisms within the brain. For example, Destrez et al. (2012) investigated the effect of diazepam (commonly known as Valium) on the judgment biases of female lambs. Diazepam is a benzodiazepine known to reduce fearfulness (Dantzer, 1977). The researchers found that lambs that had been administered diazepam displayed shorter approach times when presented with an ambiguous location compared to the control lambs that received a saline injection. They concluded that diazepam induced a greater degree of positive affect and, thus, a more positive response to the ambiguous cues relative to that of the control lambs. Similarly, this anxiolytic effect after receiving palatable food rewards was also found to be enhanced when sheep were administered a morphine injection (Verbeek et al., 2014).

A potentially important hormone for the onset and maintenance of a positive cognitive bias is oxytocin (OT). OT shows significant binding in the limbic system (Heinrichs et al., 2009) and has been shown to decrease anxiety and stress responses in several species (rats, Slattery and Neumann, 2010; Ayers et al., 2011; squirrel monkeys, Parker et al., 2005; humans, Tops et al., 2013). It was predicted that increasing OT levels would lead to shorter latency of approach on ambiguous trials in rats because the anxiolytic effects of OT should result in a higher level of positive affect (i.e., optimism). If rats approach a novel or ambiguous stimulus as fast as or faster than they approach a known rewarded stimulus (i.e., a goal pot), it may indicate that rats expect a food reward or a positive outcome. Conversely, if they approach the novel or ambiguous goal pot as slowly as (or slower than) they approach a goal pot known to contain an aversive stimulus, it may indicate that the rats have a pessimistic expectation of the outcome (expecting that there will be a mildly aversive outcome, such as an absence of food or an aversive stimulus). For these experiments “optimism” is defined as responding to an ambiguous item in the same manner as to items previously associated with reward, and “pessimism” is defined as responding to an ambiguous item in the same manner as to items previously associated with no reward or an aversive stimulus. Rats make particularly interesting subjects for such research given that they often show neophobic responses to novel stimuli (Barnett, 1958; Mitchell, 1976), although recent literature has called this conclusion into question (Ennaceur et al., 2009). We proposed that OT may increase the speed with which potentially otherwise neophobic rats would approach novel cue combinations.

Effects of OT on learning and memory in rats appear to be extremely dose-dependent. Kovács et al. (1979) found that OT reduced the retention of a passive avoidance response to a foot shock but only when OT was delivered directly into certain areas of the brain (e.g., the hippocampus). Surprisingly, these researchers also found that OT directly injected into the dorsal septal nuclei facilitated memory consolidation. Further complicating the picture, Popik et al. (1992) found that low doses of OT (0.09–6 ng/kg) given subcutaneously dose-dependently facilitated social recognition whereas higher doses (24 ng/kg) of OT attenuated memory. Whereas some studies have shown that OT in certain doses and administered in certain ways can result in an attenuation of memory and learning, low doses, similar to those used in this study, may actually improve memory function. Given existing research on the effects of OT on memory and learning, negative effects of OT on memory in this study were not anticipated. This is important in order to demonstrate that a bias toward responding to the ambiguous stimulus was not due to forgetting the value of the trained reward or aversive stimuli.

The purpose of this study was to use a modified conditioned place preference (CPP) apparatus to test the effects of OT on cognitive bias measured as response to ambiguous place cues. The CPP apparatus has been utilized previously in studies on the motivational properties of OT, with researchers finding that OT produced place preferences when administered repeatedly in specific environments due to its rewarding properties (Liberzon et al., 1997). The CPP apparatus lent itself easily to a study of cognitive bias due to the ability to manipulate the combination of cues in the opposing larger compartments.

Because of the large variety of doses and administration techniques described in the literature, and a gap in published literature concerning the effects of hormones on cognitive biases, we also conducted additional tests to assess the effectiveness of the dose used in Experiment 1 in a modified place preference (Experiment 2) and alcohol consumption test (Experiment 3).

Liberzon et al. (1997) found that rats that were repeatedly administered 6 mg/kg OT when placed in a non-preferred compartment reversed their preferences whereas those administered saline injections displayed a lack of preference. Based on these findings, it could be assumed that if the dose used in Experiment 1 was also effective, it would also have induced a place preference. If the dose used was effective in reducing stress or anxiety levels but did not result in a change in cognitive bias or place preference, we would expect that the dose might influence other measurable behaviors such as voluntary consumption of an anxiolytic. We decided to investigate the possible effects of OT on alcohol for a larger range of doses (0.0, 0.001, 0.01, and 0.1 mg/kg) to try and pinpoint an effective dose.

## Experiment 1

### Methods

#### Subjects

Sixteen juvenile male Sprague-Dawley rats took part in the experiment. All rats were approximately 200–300 g at the onset of training. Eight young adult rats were randomly assigned to the OT treatment and eight assigned to the saline treatment. The rats were tested in consecutive groups of eight (four in each treatment). The protocol was approved by Institutional Animal Care and Use Committee and the rats were treated in accordance with the Guide for the Care and Use of Laboratory Animals (National Research Council, 2011).

Subjects were housed individually in standard cages with water provided *ad libitum*. Enrichment was provided in the form of Iso-Blox and Nylabones (Harlan Teklad, Chicago, IL, USA) which are used by the rats to engage in foraging activity and to shred for bedding. Rats were housed individually for two reasons. First, individual housing facilitated monitoring of home-cage food and fluid consumption. Second, social housing potentially may have interacted with the “social bonding” hormone OT to alter cognitive bias in the behavioral paradigm. Ågren et al. (1997) found that the oxytocinergic mechanism of rats were activated if their cagemate was injected with OT. A single housing arrangement minimized the diffusion of OT via olfactory mechanisms. As OT affects social bonding and relationships (Uvnäs-Moberg, 1998), it was important to house the subjects individually following OT exposure to ensure that OT did not alter social interactions and learning as the animals progressed through training. For Group 1, food was provided *ad libitum* outside of training and testing days. On training and testing days, rats were provided access food only after training or testing had concluded to motivate the rats by incentivizing the food pellets (Bio-Serv #F0021, Frenchtown, NJ, USA). Meal-duration restricted rats had temporary restricted food access rather than relying on a reduction of body weight (cf. Baker et al., 2012). Unfortunately, this method unintentionally motivated the rats to consume the unpalatable pellets (quinine hydrochloride soaked pellets, which are known to have a bitter and unpalatable taste) provided on the aversive trials, as well as the palatable food on reward trials. To ensure that the rats learned to discriminate between trial types, the procedure was modified. For Group 2, food was provided *ad libitum* at all times. Because these rats were not food restricted before training or testing, they were not motivated to consume the unpalatable pellets. To maintain motivation to eat the palatable food, this group was trained using highly preferred food items (Froot Loop^®^ fragments) as the palatable food item.

#### Materials

All test sessions were recorded with a JVC^®^ video camera. Unique wall and floor combinations were achieved using transferrable, reversible floor covers made from Chop Chop^®^ flexible cutting mats. Plastic food cups were affixed inside the compartments.

#### Apparatus

**FIGURE 1:**
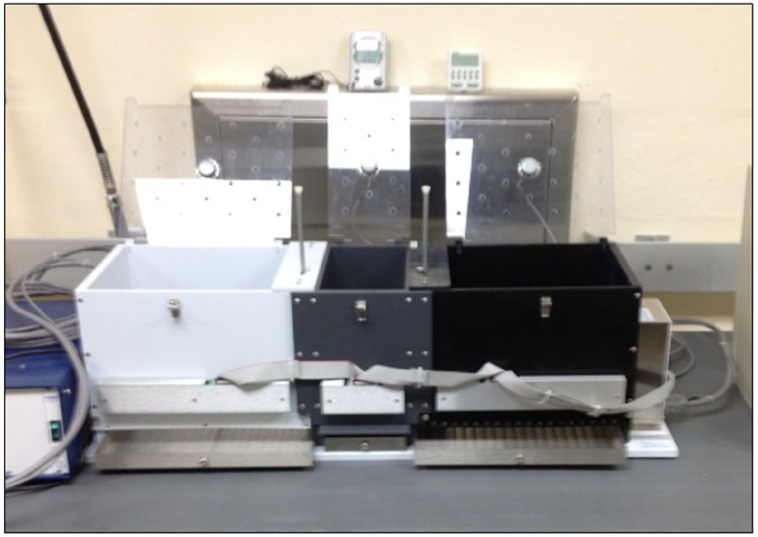
**Conditioned place preference apparatus**.

The CPP apparatus was a standard three-compartment design (Med Associates, St. Albans, VT, USA) and was housed in a separate testing room. The apparatus consisted of three compartments, with two equally-sized compartments and a central gray compartment (with manual guillotine doors), which acted as the starting chamber. Because CPP paradigms investigate preference for distinct locations, there are discriminable cues built into the CPP apparatus. In the CPP apparatus used in this study, there were unique wall colors and flooring materials in each compartment (see Figure 1).

The larger place preference compartments had distinct wall colors (black and white). In order to create a second set of place cues that could be manipulated to create different place cue combinations, differently textured flooring (smooth versus textured) was placed in the compartments. The rats’ orientation to other landmark cues in the testing room remained constant. Because the floors were interchangeable, we were able to manipulate the wall and flooring combinations to create ambiguous cue combinations. Within each of the large preference compartments a goal pot was placed near the wall farthest from the guillotine door. The latency of approach to the goal pot and to enter a compartment from the time they were placed in the starting chamber was recorded (cf. Subiah et al., 2012).

#### Treatments

Similar to Ayers et al. (2011), before beginning testing sessions, rats were given a subcutaneous injection. Injections consisted of either 0.001 mg/kg OT or saline vehicle administered subcutaneously into the scruff of the neck. Grippo et al. (2012) found that OT administered subcutaneously attenuated the autonomic stress response to social isolation even though the dose used failed to elicit behavioral changes. Additionally, Uvnäs-Moberg et al. (1994) found that low doses of OT (0.001–004 g/kg) decreased peripheral locomotor activity in an open field test, a possible indication of the anxiolytic effect of OT at such low doses. They also found that high doses (0.25–1 g/kg) had clear sedation effects. Due to the amount of time necessary for absorption to occur after injections and the short half-life of OT (Ginsburg and Smith, 1959), injections were given 5 min before testing. Dosage was chosen based on similarly small doses and administration techniques (Missig et al., 2010) that were found to affect anxiety related behavior, keeping in mind that larger doses (at least those administered directly into the brain) can adversely affect memory (Kovács et al., 1979).

#### Habituation

Before beginning the training, rats were given three habituation sessions in the apparatus. Once placed in the center compartment, the rats were given 2 min to acclimate before the guillotine doors were opened and they were given 3 min to explore the larger compartments (cf. Burman et al., 2009). Rats were placed into the center compartment of the apparatus facing the back of the apparatus. For both groups, the larger compartments did not contain any pellets to avoid the rats forming any associations between the larger compartments and food rewards.

To streamline the training process, Group 2 was also given time to habituate to the goal pots and learn to retrieve the food placed within them. Immediately following the apparatus habituation, the rats were placed into an empty cage in which a spare goal pot had been affixed. Within the goal pot there was a single Froot Loop^®^. Rats received a minimum of two goal pot habituation sessions lasting 5 min. If they were unsuccessful after two attempts (*N* = 2) they were given a maximum of two additional open ended sessions until they successfully retrieved the Froot Loop^®^.

Additionally, all rats were administered three mock injections prior to all testing in which they were restrained and injected with saline in order to habituate them to the injection procedure.

#### Training

Rats participated in a minimum of two 12-trial training sessions (a maximum of five) during which they learned to discriminate between the reward compartment (which always contained palatable food) and the aversive compartment (which always contained unpalatable food). For each trial, only the door to the aversive or reward compartment was open. The rats were trained to distinguish between two chambers: a white compartment with smooth flooring and a black compartment with textured flooring. For half of the rats in each treatment, the white compartment combination was designated the reward combination while the black compartment combination was designated the aversive combination; for the other half, these cues were reversed. Training sessions consisted of six reward and six aversive combinations presented in randomized order with no more than three consecutive trials of the same type.

For each trial, the rat was placed in the center compartment of the apparatus for a 2 min acclimation period after which one of the guillotine doors was raised, allowing access to either the reward compartment or the aversive compartment. On reward training trials, the rats had access to the reward compartment, in which the goal pot contained five palatable food pellets for Group 1 or half of a Froot Loop^®^ for Group 2. On aversive training trials, the rats had access to the aversive compartment only, in which the goal pot contained only one unpalatable food pellet that had been soaked in 0.15% quinine hydrochloride (Sigma-Aldrich, St. Louis, MO, USA) solution for Group 1 and 0.30% quinine hydrochloride solution for Group 2 (Burman et al., 2009). The strength of the solution was increased for Group 2 to further motivate the rats to avoid eating the quinine hydrochloride pellet. After the door was opened, the time it took for the rats to contact the goal pot was recorded. Contact was defined as physically touching the pot or sniffing the pot for 2 s or more.

After an initial open-ended trial, the rats in Group 1 were given only 2 min to reach the goal pot before the rat was again placed in the center compartment for another inter-trial interval of 2 min (Burman et al., 2009). For Group 2, the trial duration was shortened by 30 s after observing that 2 min for the rats in Group 1 was more than necessary for the rats to complete the task. When the rats’ average approach latency for the reward trials within a session was at least 5 s shorter than the average approach latency on aversive trials within the same session, the rats had passed criterion for entering the testing phase. The CPP apparatus was wiped down with 70% alcohol solution at the conclusion of each training session to prevent olfactory information from being passed between animals (Burman et al., 2009).

#### Testing

After meeting criterion, rats participated in one testing session per day for two consecutive days. The testing sessions consisted of eight 120 s (Group 1) or 90 s (Group 2) trials made up of three reward trials, three aversive trials, and two ambiguous trials. Each rat was tested on each of the two ambiguous cue combinations (i.e., the white compartment and textured floor combination and the black wall and smooth floor combination) only once in each session to avoid forming associations between the cues and specific outcomes. The ambiguous trials occurred on the third and sixth trials of each session and the intervening trials were randomly distributed between reward and aversive trials with no more than two trials of a type happening consecutively (excluding ambiguous trials). Trials were counterbalanced within subjects so that there were an equal number of reward and aversive trials preceding an ambiguous trial across subjects. The order in which the ambiguous conditions were presented was also balanced across sessions to control for any order effects.

Between trials the large compartment combinations were manipulated by removing and replacing the floor covers to transition to the combinations needed for the next trial. The goal pots in the ambiguous conditions were unbaited (Burman et al., 2009). In the reward and aversive trials, rats were allowed to eat the available pellets until the conclusion of the trial, at which time the rat was removed from the apparatus. The CPP apparatus was wiped down with a 70% alcohol solution between each trial to remove any olfactory information left behind from the previous trial. Latency to approach the goal pots in the ambiguous compartments was averaged across the two trials for each rat.

#### Data Analysis

Latency to approach was analyzed using Mixed Model ANOVAs with condition (ambiguous, reward, and aversive) as the within-subject factor and treatment (OT or saline) as the between-subjects factor using SPSS 17.

### Drugs

The OT used was purchased from Bachem Americas Inc., Torrance, CA, USA. Upon arrival, the OT was reconstituted in 0.9% saline (to 0.01 mg/ml) and then stored at –20°C in 1 ml aliquots until the day of testing. Before the testing session, an aliquot of OT was thawed and diluted with 0.9% saline to the appropriate concentration of 0.001 mg/ml to allow an injection volume of 1 ml/kg for each rat.

### Results

Data from two rats (both in Group 1) were dropped prior to analysis; one because of failure to discriminate between trial types during training and another because of reluctance to exit the center compartment during both training and testing. A Pearson’s correlation coefficient was computed to assess reliability between a naive observer working from video and the experimenter watching in real time with regard to the rats’ latency to contact the pot. There was a strong positive correlation between the coded latencies (*r* = 0.758, *N* = 14, *p* = 0.002). Given the strong correlation between the observer codings, all reported analyses are based on data from the naïve coder.

A mixed model ANOVA with condition (ambiguous, reward, and aversive) as the within-subject factor and group (Group 1 and Group 2) as a between-subjects factor was then conducted to determine whether there was an effect of group on latency to contact the goal pot. The effect of group on the latency to approach the goal pot was non-significant (*F*_2,24_ = 0.911, *p* = 0.416, observed power = 0.189, ${\text{η}}_{p}^{2}$ = 0.071). Because there was no significant difference in the latencies of the rats to contact the pot between groups, data was collapsed across the two groups in all subsequent analyses for a total of 14 rats^1^.

**FIGURE 2:**
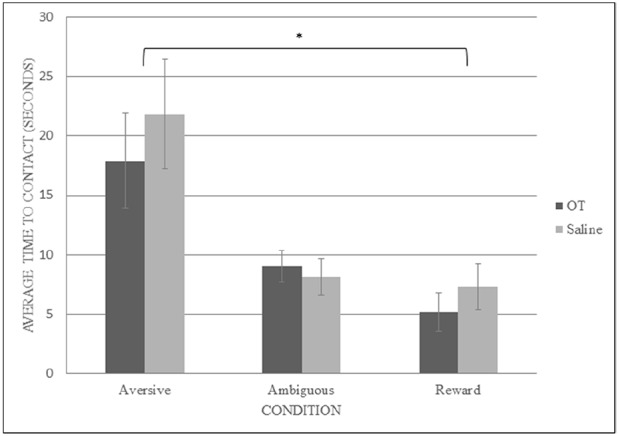
Contact latency: OT vs saline treatments *p < 0.001.

To determine the effect of OT on the latency to contact the goal pot, a mixed model ANOVA was again conducted, this time with treatment (OT or saline) as the between-subjects variable and condition (ambiguous, reward, and aversive) as the within-subjects variable. There was a significant main effect of condition type on latency to contact the pot (*F*_2,24_ = 20.901, *p* < 0.001, observed power = 1.000, ${\text{η}}_{p}^{2}$ = 0.635). We were particularly interested in whether rats responded to ambiguous cues as they did to aversive or reward conditions, so planned simple contrasts with comparisons of aversive and reward to ambiguous conditions were conducted and revealed a significant difference between the contact latencies for ambiguous conditions (*M* = 8.578, SEM = 1.024) and aversive conditions (*M* = 19.865, SEM = 3.061; *F*_2,24_ = 20.808, *p* = 0.001, observed power = 0.987, ${\text{η}}_{p}^{2}$ = 0.634), but not between ambiguous conditions and reward conditions (*M* = 6.238, SEM = 1.253; *F*_2,24_ = 3.624, *p* = 0.081, observed power = 0.418, ${\text{η}}_{p}^{2}$ = 0.232). This result suggests that the rats learned to discriminate between the trained cue conditions (reward and aversive) and that they treated the ambiguous conditions in a similar fashion as they did the reward conditions regardless of treatment (see Figure 2). There was no significant interaction between treatment and condition (*F*_2,24_ = 0.590, *p* = 0.562, observed power = 0.137, ${\text{η}}_{p}^{2}$ = 0.047). There was no significant main effect of treatment on contact latency (*F*_1,12_ = 0.322, *p* = 0.581, observed power = 0.082, ${\text{η}}_{p}^{2}$ = 0.026).

Because the combination of white walls and smooth floor was the trained reward cue for half of the rats whereas the other half learned the combination of black walls and textured floor as the reward cue combination, a mixed model ANOVA with the two ambiguous cue conditions as the within-subject factors and the trained cue combination groups as the between-subjects factor was conducted to ensure that the rats’ contact latencies were not an effect of the cue combinations they were trained on. This test was conducted to ensure that the rats were not attending to only one aspect of the cue combination (wall color, floor texture, or even external spatial cues), which may have caused them to respond to the ambiguous conditions as if they were equivalent to the trained cues (not because they were in positive states, but because they did not perceive the distinction between the conditions). Likewise, they might have perceived the ambiguous condition to be equivalent to the aversive condition, depending on which cues they prioritized during training. However, there was no significant interaction between the two ambiguous trial types and the trained reward cue combination for latency to approach (*F*_1,12_ = 0.133, *p* = 0.722, observed power = 0.063, ${\text{η}}_{p}^{2}$ = 0.011), negating this concern.

A mixed model ANOVA with both session (the last training session and the average of the testing sessions) and condition (aversive and reward) as the within-subjects factors, and treatment (OT or saline) as the between-subjects factor was conducted in order to verify whether OT changed the way that the rats responded to the reward and aversive cue combinations during the testing phase compared to their performance on their last training session. There was no significant interaction of session, condition, and treatment on the contact latencies (*F*_1,12_ = 0.199, *p* = 0.663, observed power = 0.608, ${\text{η}}_{p}^{2}$ = 0.016). There was no significant interaction of session and condition on contact latency (*F*_1,12_ = 3.043, *p* = 0.107, observed power = 0.362, ${\text{η}}_{p}^{2}$ = 0.202). There was a significant main effect of condition on the latency to contact the goal pot (*F*_1,12_ = 23.431, *p* < 0.001, observed power = 0.993, ${\text{η}}_{p}^{2}$ = 0.661) with the latency on aversive conditions (*M* = 31.724, SEM = 5.286) significantly longer than for reward conditions (*M* = 7.588, SEM = 1.345). There was also a significant main effect of session type (*F*_1,12_ = 5.908, *p* = 0.032, observed power = 0.608, ${\text{η}}_{p}^{2}$ = 0.330) in that rats responded more quickly overall on testing (*M* = 13.051, SEM = 1.889) compared to training (*M* = 26.260, SEM = 5.340), which may have been due to the increase in the cues’ salience as the rats progressed through the training and testing sessions. There was no significant main effect of treatment (*F*_1,12_ = 0.020, *p* = 0.889, observed power = 0.052, ${\text{η}}_{p}^{2}$ = 0.002).

### Discussion

Although there were no differences in contact latencies between treatment groups, the rats were found to display what would be considered positive cognitive bias (optimism). This optimism may have resulted from the anticipation of food rewards and the opportunity to explore outside of their home cage. One possible explanation for the lack of observed effect of OT is that the dose used was ineffective. We anticipated that a significant difference in latencies between the groups would confirm the effectiveness of the dose. In the absence of such an effect, it became necessary to evaluate the effectiveness of the dose in other contexts as a manipulation check. One significant finding, that the rats traveled faster toward the goal pot on test trials compared to training trials could have been due to an increase in the salience of the cues or in a reduction of neophobia as the rats became more familiar with the testing apparatus throughout the experiment.

## Experiment 2: Conditioned Place Preference and Oxytocin

One way of assessing the effectiveness of the dose used in Experiment 1 was to use a modified CPP paradigm. Doses that would elicit a place preference would theoretically also be capable of influencing cognitive bias. CPP tests have also been validated as measures of affect in non-human animals. Millot et al. (2014) validated the use of behavioral as well as physiological indicators of affective state during a CPP task for gilthead sea bream fish.

Based on the findings of Liberzon et al. (1997), we investigated whether the dose used in the current study, which was much smaller than that used by Liberzon et al. (1997), could cause a reversal of an initial preference for a chamber in a CPP apparatus. Note that the smaller dose was used here to alleviate concerns with the potential for OT to interrupt memory.

### Methods

#### Subjects

The same 16 subjects that participated in Experiment 1 also participated in Phase 1 of Experiment 2. For Phase 2, an experimentally naive group of six young adult male Sprague-Dawley rats, all between 250 and 350 g, participated in a classic CPP experiment.

#### Testing

***Phase 1***

At the conclusion of the testing phase for each rat in the cognitive bias experiment, a variation on a CPP test was conducted in order to test the effectiveness of the OT dose used. Rats were injected with either saline or OT 5 min prior to the testing session. During the testing session, the rat was placed in the gray center compartment and was given a 5 min acclimation period immediately followed by a 15 min exploration period in which both guillotine doors were opened. If effective, rats given OT should spend more time in the trained aversive compartment compared to the rats administered saline due to the anxiolytic effects of OT.

Group 1 rats were administered two CPP tests. For the first CPP test, the rats were again administered 0.001 mg/kg OT or saline. For the second CPP test, the OT dose was increased to 0.01 mg/kg OT. The rats in Group 2 were administered a single CPP test. The OT dose used was again 0.001 mg/kg OT. For each test, the rats were assigned to the same treatment groups as in Experiment 1.

***Phase 2***

Due to the ambiguous results of both the cognitive bias experiments and the first phase of the CPP follow-up, it was necessary to perform more rigorous testing to determine an effective dose of OT. On the first day of testing, rats were given a pre-training session in which the rat was placed into the gray compartment for a 1 min acclimation period after which both guillotine doors were raised and the rat was free to explore all three chambers for 15 min. This test assessed the innate preferences for chambers based on duration within the different chambers. In this case, all six rats spent more time in the black chamber than the white chamber. The pre-training day was immediately followed by four consecutive training days, which consisted of one training session per day. Five minutes before the training sessions, each rat was injected with OT. After the injection, the rats were placed into their least preferred chamber (e.g., the white chamber) for 20 min. Unlike in traditional CPP experiments, the rats were not given sham injections in the black chamber during the training phase and as a consequence, were only exposed to the black chamber on the pre-training and post-training test sessions.

For Phase 2, the CPP test was administered twice. For the first test, all six rats were administered OT doses of 0.001 mg/kg. The test was then repeated a week later using doses of 0.01 mg/kg of OT for all six rats.

### Results

#### Phase 1

For each of the CPP follow up tests, a repeated-measures ANOVA on time spent in the compartment with compartment (center, reward, and aversive) as a within-subject factor and treatment (OT, saline) as a between-subjects factor was conducted. After testing Group 1, two CPP tests, one using 0.01 mg/kg OT and one using 0.001 mg/kg OT were conducted. For the 0.001 mg/kg dose there were no significant main effects of compartment (center, aversive, or reward; *F*_2,12_ = 2.399, *p* = 0.133, observed power = 0.391, ${\text{η}}_{p}^{2}$ = 0.286) or treatment (*F*_1,6_ = 0.000, *p* = 1.000, observed power = 0.050, ${\text{η}}_{p}^{2}$ = 0.000) on the amount of time spent in the compartments, and there was no significant interaction of the compartment and treatment (*F*_2,12_ = 0.608, *p* = 0.560, observed power = 0.129, ${\text{η}}_{p}^{2}$ = 0.092). When tested again using 0.01 mg/kg doses, there was a significant main effect of compartment (*F*_2,12_ = 8.766, *p* = 0.005, observed power = 0.917, ${\text{η}}_{p}^{2}$ = 0.594), with simple contrasts indicating that the rats spent significantly more time in the center compartment (*M* = 485.808, SD = 49.115) compared to the reward compartment (*M* = 235.079, SD = 48.770; *F*_1,6_ = 7.588, *p* = 0.033, observed power = 0.634, ${\text{η}}_{p}^{2}$ = 0.558). There was no significant main effect of treatment (*F*_1,6_ = 0.833, *p* = 0.397, observed power = 0.121, ${\text{η}}_{p}^{2}$ = 0.122) and no significant interaction between compartment and treatment (*F*_2,12_ = 0.707, *p* = 0.513, observed power = 0.142, ${\text{η}}_{p}^{2}$ = 0.105). For Group 2 only a single CPP test was conducted, again using 0.001 mg/kg OT. There was again no significant interaction between compartment type and treatment with regard to time spent (*F*_2,12_ = 0.260, *p* = 0.775, observed power = 0.082, ${\text{η}}_{p}^{2}$ = 0.042). There was no significant main effect of treatment (*F*_1,6_ = 0.090, *p* = 0.774, observed power = 0.058, ${\text{η}}_{p}^{2}$ = 0.015). This group also displayed no significant main effects of compartment type on time spent in the compartments (*F*_2,12_ = 3.132, *p* = 0.080, observed power = 0.492, ${\text{η}}_{p}^{2}$ = 0.343). The results of all of these CPP tests show that, regardless of dose, there was no difference in how saline and OT dosed rats budgeted their time between the three compartments.

#### Phase 2

To analyze the results from the modified CPP tests, a repeated-measures ANOVA was conducted with both session (pre-training and post-training) and compartment (gray, white, and black) as the within-subject factors to determine the effects of the CPP procedure on the amount of time spent in the compartments. When the rats were given 0.001 mg/kg OT, there was no significant main effect of session on how the rats budgeted their time in the apparatus (*F*_1,5_ = 1.000, *p* = 0.363, observed power = 0.130, ${\text{η}}_{p}^{2}$ = 0.167). There was a main effect of compartment on time spent (*F*_2,10_ = 4.541, *p* = 0.040, observed power = 0.633, ${\text{η}}_{p}^{2}$ = 0.476), with rats spending significantly more time in the black chamber compared to the white chamber. There was a significant interaction of session type and compartment (*F*_2,10_ = 7.699, *p* = 0.009, observed power = 0.859, ${\text{η}}_{p}^{2}$ = 0.606).

**FIGURE 3:**
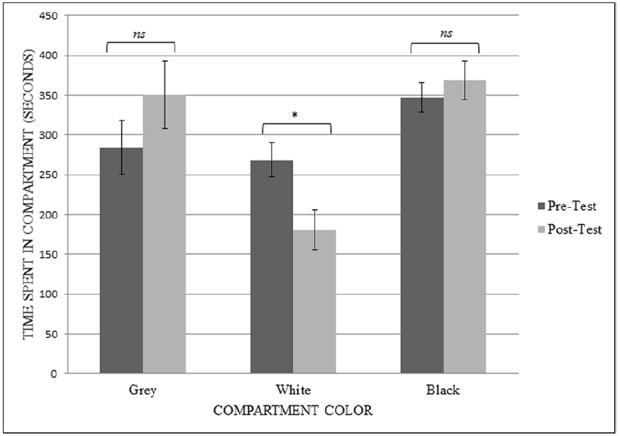
Experiment 2, Phase 2: interaction between session type and compartment type *p = 0.04.

To evaluate the interaction, paired-samples *t*-tests were performed to compare the amount of time spent in each specific compartment before and after the training sessions. When given doses of 0.001 mg/kg OT, there was a significant difference in the time spent in the white chamber (which all rats originally showed the least preference for) after the rats had undergone training using 0.001 mg/kg of OT (*t*_5_ = 7.867, *p* = 0.001). Contrary to predictions, they spent less time in the white chamber in the post-test session (*M* = 180.687, SD = 60.32) than they did in the pre-test session (*M* = 268.477, SD = 51.86). There was no significant difference between how much time they spent in the gray chamber (*t*_5_ = –2.068, *p* = 0.093) or in the black chamber (*t*_5_ = –0.980, *p* = 0.372) after the training sessions in the white chamber (see Figure 3).

For the 0.01 mg/kg dose CPP test, a repeated-measures ANOVA revealed no significant interaction between session type and compartment (*F*_2,10_ = 3.044, *p* = 0.093, observed power = 0.460, ${\text{η}}_{p}^{2}$ = 0.378) and no significant main effect of session type (*F*_1,5_ = 0.172, *p* = 0.695, observed power = 0.064, ${\text{η}}_{p}^{2}$ = 0.033). However, there was a main effect of compartment on the time spent in the compartments (*F*_2,10_ = 9.434, *p* = 0.005, observed power = 0.922, ${\text{η}}_{p}^{2}$ = 0.654) with simple contrasts indicating that the rats spent significantly more time in black (*M* = 347.583, SD = 29.11) compared to the white (*M* = 198.667, SD = 12.995) compartment (*F*_1,5_ = 15.609, *p* = 0.011, observed power = 0.880, ${\text{η}}_{p}^{2}$ = 0.757). The simple contrast between time spent in black and time spent in gray was not significant (*F*_1,5_ = 0.014, *p* = 0.910, observed power = 0.051, ${\text{η}}_{p}^{2}$ = 0.003).

### Discussion

In Experiment 2, we attempted to find an appropriate dose for a replication of Experiment 1, but findings suggest that neither dose of OT was having the hypothesized effect on behavior. This suggests that the null results regarding treatment obtained in the cognitive bias testing could have resulted from an ineffective dose of OT.

The rats in the CPP tests displayed a clear preference for the black chamber during the pre-training session and they did not show a stronger preference for black after the post-training session. The only significant change between pre and post-training was that the rats spent less time in the white chamber. The doses of both 0.001 mg/kg or 0.01 mg/kg of OT failed to increase the amount of time the rats spent in the aversive conditions compared to those rats dosed with saline. Because injections of OT prior to placement in the non-preferred chamber also failed to reverse the rats’ initial preferences, it is clear that these doses were ineffective with regard to at least this aspect of behavior. Based on these findings, it would appear that the OT dose was not successful in inducing a place preference.

Problems with the doses may have stemmed from differences in effects of OT based on the combination of administration techniques and dose strength. In the literature there are examples of a wide range of doses administered using a variety of methods, including subcutaneous injections (Missig et al., 2010) and intracranial injections (Kovács et al., 1979). It may be that the effect of OT on affect is dose-dependent and also dependent on the manner of delivery. More research is needed to investigate what forms of OT administration may be most likely to facilitate changes in optimism or pessimism. Until this area has been explored more thoroughly, it would be premature to assume that OT has no effect on the development and maintenance of certain cognitive biases.

It is also important to note that the procedure used here deviated from standard CPP protocols. Unlike in most other CPP designs, rats in this study were not given vehicle only injections when in the preferred chamber during the training portion of the study. This resulted in less time spent in the more preferred chamber during the training, potentially making it more interesting at the second test. This, in turn, may have resulted in the rats associating the less preferred chamber with injections, making it difficult to observe a reversal of preference, even with the administration of OT. However, we do not think this methodological deviation negated the possibility of finding an OT effect where one truly existed, given that the rats did not significantly increase the time spent in the black chamber and spent slightly more time (though not significantly more time) in the center gray chamber in the post-training session. Theoretically, if the OT had produced an anxiolytic effect, we would have expected the rats to spend more time outside of the gray compartment exploring the apparatus. Furthermore, the rats giving the higher dose of OT did not spend less time in the white chamber following the injections.

## Experiment 3: Oxytocin and Alcohol Consumption

If the OT doses used in Experiments 1 and 2 were effective in reducing stress or anxiety levels, despite having no effect on cognitive bias or place preference, then we would expect that the doses would influence behavior in other predicable ways. For instance, Zhou et al. (2015) found that lower doses (0.3 mg/kg for male rats; 0.3, 1, and 3 mg/kg for female rats) of OT decreased sucrose consumption. OT has also been found to decrease methamphetamine consumption and seeking behaviors in rats (Carson et al., 2010). If the doses used in previous experiments functioned as anxiolytics, then it is possible that they should reduce the consumption of alcohol given that alcohol consumption serves to reduce stress or anxiety levels. We decided to investigate the possible effects of OT on alcohol for a larger range of doses (0.0, 0.001, 0.01, and 0.1 mg/kg) to try and pinpoint an effective dose.

### Methods

#### Subjects

Subjects were 20 adult male Sprague-Dawley rats approximately 400–450 g and 125 days old at the beginning of the experiment. The rats had a behavioral history of alcohol consumption, but they had not previously participated in a CPP task or cognitive bias testing.

#### Apparatus

Rats were tested using standard operant chambers (30.5 cm × 24.2 cm × 29.2 cm) containing two levers located 7 cm from the floor on the right wall (Med Associates, St. Albans, VT, USA). Between the two levers was a receptacle cup approximately 3 cm from the floor. The cup was supplied with reinforcing fluid from a syringe pump connected to the cup by 18 gage stainless steel tubing.

#### Treatment

Subjects were administered subcutaneous injections of 0.0, 0.001, 0.01, and 0.1 mg/kg OT during different test sessions. OT was dissolved in 0.9% saline and stored as aliquots at –20°C. On the day of the first testing session, aliquots were diluted in 0.9% saline to concentrations of 0.001, 0.01, and 0.1 mg/kg to allow administration of 1 ml/kg volume. Unused volumes of the OT solution were refrigerated and used on the two subsequent injection days (the manufacturer indicated that reconstituted OT was stable for 1 week when refrigerated). OT was administered subcutaneously into the scruff of the neck 5 min before the operant session. Each rat received all of the treatments in a random order, except that the saline vehicle treatment was always administered first. Before training began, all of the rats were administered three mock injections of saline to habituate them to the injection procedure.

#### Training

All rats had been trained to lever-press for alcohol in previous studies. During response periods, rats earned fluid alcohol reinforcers of 0.1 ml by lever-pressing on a continuous reinforcement schedule. The reinforcer was 10% ethanol (wt/vol), 2% sucrose and 0.1% saccharin mixed in distilled water.

#### Testing

Oxytocin testing took place over 8 days, with one OT session every other day, between which the rats took part in sessions in which no injections took place in order to establish a baseline of alcohol consumption. For the first testing session, all rats were given a saline vehicle injection (0.0 mg/kg OT). For the three following testing sessions, all rats were administered injections of one of three concentrations of OT (0.001, 0.01, or 0.1 mg/kg). The dose order was randomized using a Latin square matrix. Rats received subcutaneous injections and were then placed into the operant chambers for a 5 min waiting period during which the chamber was dark. After the waiting period, the 30 min testing (response) session began in which the rats were able to lever-press for ethanol. At the conclusion of the testing session, the rat was removed from the chamber and any amount of fluid remaining in the receptacle cup was measured and subtracted from the measured amount removed from the syringe pump. These measurements were used to calculate the ethanol intake (g/kg) for each rat.

### Results

A repeated-measures ANOVA with the treatment (baseline, saline, 0.001, 0.01, and 0.1 mg/kg OT) as the within-subject factor was conducted on consumption rates. There was no significant effect of treatment on alcohol consumption (*F*_1,19_ = 1.014, *p* = 0.406, observed power = 0.306, ${\text{η}}_{p}^{2}$ = 0.051). A second repeated-measures ANOVA was conducted to analyze the effect of treatment on the number of responses (lever-pressing). There was no significant effect of treatment on the number of responses (*F*_1,19_ = 1.256, *p* = 0.295, observed power = 0.375, ${\text{η}}_{p}^{2}$ = 0.062).

### Discussion

If one of the doses of OT was found to affect alcohol consumption in these rats, it would have provided evidence that the OT dose was pharmacologically active and the cognitive bias experiment described earlier could be replicated using pharmacologically active doses. We predicted that an effective dose of OT might cause a decrease in alcohol consumption because rats may significantly increase their alcohol consumption as a way of coping with the stressor of experiencing restraint (Lynch et al., 1999). Previous research has found that OT administered both intracerebroventricularly (1–10 μg/rat) and intraperitoneally (375–3,000 μg/kg) dose dependently inhibited fluid intake (Arletti et al., 1990). If the OT doses used in Experiment 3 had been effective, we could have expected a decrease in alcohol consumption due to the anxiolytic effects of OT. Unfortunately, the OT doses in this experiment failed to decrease alcohol consumption in rats. Thus, these OT doses may not be pharmacologically active doses or the behavioral paradigm requires greater sensitivity to observe the effects of OT.

## General Discussion

Although this was the first study to specifically address the potential effects of OT on cognitive bias, other research has addressed the effects of OT on anxiety levels (Ayers et al., 2011), which is closely linked to negative cognitive bias. For instance, Burman et al. (2009) found that anxiety caused by high intensity light exposure was correlated with a significant reduction in response latency for rats. OT has been shown to attenuate anxiety and reduce the response of the HPA (hypothalamic-pituitary-adrenal) axis to stressors. OT-deficient mice display more anxiety-related behaviors as compared to control mice when exposed to psychological stressors (Amico et al., 2004). Missig et al. (2010) found that doses of 0.1 and 0.01 μg OT delivered subcutaneously were able to reduce various behaviors related to background anxiety such as acoustic startle response. Because OT has been shown to influence anxiety related behaviors, it would follow that OT may also influence related phenomena such as negative cognitive biases.

Although the results of Experiment 1 did not show a difference in cognitive bias (as indicated by contact latency) between the OT treated and saline treated rats, all of the rats did appear to exhibit positive cognitive bias when faced with ambiguous stimuli. The rats approached the goal pot in the ambiguous condition almost as quickly as they did the reward condition regardless of the OT dose. The benefit of incorporating the CPP apparatus into a cognitive bias test of this type is that it utilizes cues for the ambiguous trials which are not intermediate on a spectrum between the reward and aversive cues. Many cognitive bias tasks make use of intermediate ambiguous cues (e.g., shades of gray when the trained stimuli are black and white; Bateson and Matheson, 2007; Burman et al., 2011; Bethell et al., 2012; Keen et al., 2014). In essence the ambiguous testing phase then becomes a categorization task. By utilizing combinations of cues which provide conflicting information, there is less of a chance that the subjects will attempt to solve the task by responding according to which trained cue the ambiguous stimuli most resembles (e.g., categorizing dark gray with black and light gray with white).

### Limitations and Future Directions

One significant limitation of these experiments was the possibility that either the dose of OT or the administration of the dose was ineffective. There are a wide variety of doses and administration techniques outlined in the existing literature. It is possible that in order for OT to exert effects in cognitive bias paradigms, it needs to be injected directly into the brain. Before it can be concluded with certainty that this testing paradigm is ineffective, separate tests should investigate the effects of a greater range of OT doses and administration methods on various related behaviors.

Another limitation of this paradigm concerns the difference between temporary effects as a result of the experimental manipulation (such as current environmental conditions) and more chronic temperament biases. It may be that there are individual differences in chronic temperament biases, with certain individuals reacting positively or negatively to ambiguous stimuli in any situation regardless of the manipulations. Chaby et al. (2013) found that rats that experienced chronic unpredictable stress as juveniles displayed chronic negative cognitive bias in adulthood, as well as other behavioral and cognitive changes when compared to a control group. Research designs should attempt to tease apart the effects of short-term and long-term biases. One way to test for the presence of chronic temperament biases would be to test individuals in a variety of experimental manipulations and contexts to investigate whether the individual’s biases are maintained across the test conditions or whether their biases fluctuate with the changes in testing conditions (e.g., do they display positive cognitive biases after positive experiences and negative cognitive biases after negative experiences?).

A better understanding of the effects of OT on cognitive bias could allow for improved techniques and designs for animal welfare practices. Already, OT has been found to be useful as a potential biomarker of positive emotion in both dogs (Mitsui et al., 2011) and humans (Yuen et al., 2014). However, many social processes have been shown to be affected by nonapeptides such as OT in different ways depending on the sex, species, and even personality of the test subject (Goodson, 2013; Kelly and Goodson, 2014). For instance, OT is known to increase intimate partner violence in people with aggressive personality traits (DeWall et al., 2014) but is also found to facilitate pro-social behavior in rats and mice (Lukas et al., 2011). Due to the wide range of reported OT effects in the existing literature, the usefulness of OT as a possible treatment for negative cognitive biases will be highly context specific and all factors, such as species, sex, behavioral phenotype, and personality, will need to be taken into account.

In the current context, replication of this study with an appropriate dose of OT may support the use of OT as a treatment or tool in the management of some captive animals. If OT was found to be useful in influencing cognitive biases in animals, there would also be potential for the use of OT as a treatment of negative cognitive biases in humans. However, if OT continues to prove ineffective in facilitating positive biases, future research could focus on the possible influences of other hormones such as cortisol, a stress hormone, and its potential role in the onset and maintenance of negative cognitive biases. Given that intranasal OT can attenuate cortisol levels at times of social and physical stress, future research should consider the interactions of these hormones, as well as dose-dependent effects (Cardoso et al., 2013). Also pertinent would be whether findings in non-humans would hold up across species, particularly humans. The current data adds to the existing pattern of findings that will help researchers refine doses and methods to paint a clearer picture of the effects of OT on affect in at least one species.

### Conflict of Interest Statement

The authors declare that the research was conducted in the absence of any commercial or financial relationships that could be construed as a potential conflict of interest.

## Footnotes

1 A mixed model ANOVA was run for Group 1 and Group 2 separately with condition (reward, aversive, and ambiguous) as the within-subject factor and treatment (OT and saline) as the between-subject factor. There was no significant interaction of treatment and condition for Group 1 (*F*_2,8_ = 1.686, *p* = 0.245, observed power = 0.257, ${\text{η}}_{p}^{2}$ = 0.269) or Group 2 (*F*_2,12_ = 0.467, *p* = 0.638, observed power = 0.109, ${\text{η}}_{p}^{2}$ = 0.072). Both Groups showed a significant effect of condition on latency to contact the goal pot (Group 1: *F*_2,8_ = 10.265, *p* = 0.006, observed power = 0.921, ${\text{η}}_{p}^{2}$ = 0.720; Group 2: *F*_2,12_ = 12.439, *p* = 0.001, observed power = 0.980, ${\text{η}}_{p}^{2}$ = 0.675). Because the pattern of results for both Groups was the same and the OMNIBUS ANOVA indicated no effect of group on contact latencies, data was subsequently collapsed across the two groups.
